# Racial and Geographic Disparities in Colorectal Cancer Incidence and Associated County-Level Risk Factors in Mississippi, 2003–2020: An Ecological Study

**DOI:** 10.3390/cancers17020192

**Published:** 2025-01-09

**Authors:** Shamim Sarkar, Sasha McKay, Jennie L. Williams, Jaymie R. Meliker

**Affiliations:** 1 Program in Public Health, Stony Brook Medicine, Stony Brook, NY 11794, USA; mdshamim.sarkar@stonybrookmedicine.edu; 2 Department of Family, Population and Preventive Medicine, Stony Brook Medicine, Stony Brook, NY 11794, USA; jennie.williams@stonybrookmedicine.edu; 3 Department of Biochemistry, Stony Brook University, Stony Brook, NY 11794, USA; sasha.a.mckay@gmail.com; 4 Increasing Diversity in Undergraduate Cancer Biology, Education, and Research (INDUCER) Program, Stony Brook University, Stony Brook, NY 11794, USA

**Keywords:** colorectal cancer, incidence, racial disparities, food insecurity, Mississippi

## Abstract

Introduction: Colorectal cancer (CRC) is the third most commonly diagnosed cancer in the United States (U.S.). Mississippi has the highest rate of CRC incidence in the U.S. and has large populations of black and white individuals, allowing for studies of racial disparities. Methods: We conducted an ecological study using the county as the unit of analysis. CRC incidence data at the county level for black and white populations in Mississippi, covering the years 2003 to 2020, were retrieved from the Mississippi Cancer Registry. Age-adjusted incidence rate differences and their corresponding 95% confidence intervals (CIs) were then calculated for these groups. Getis–Ord Gi* hot and cold spot analysis of CRC incidence rate racial disparities was performed using ArcGIS Pro. We used global ordinary least square regression and geographically weighted regression (MGWR version 2.2) to identify factors associated with racial differences in CRC incidence rates. Results: Age-adjusted CRC incidence rate in the black population (median = 58.12/100,000 population) and in the white population (median = 46.44/100,000 population) varied by geographical area. Statistically significant racial differences in CRC incidence rates were identified in 28 counties, all of which showed higher incidence rates among the black population compared to the white population. No hot spots were detected, indicating that there were no spatial clusters of areas with pronounced racial disparities. As a post hoc analysis, after considering multicollinearity and a directed acyclic graph, a parsimonious multiple regression model showed an association (β = 0.93, 95% CI: 0.25, 1.62) indicating that a 1% increase in food insecurity was associated with a 0.93/100,000 differential increase in the black–white CRC incidence rate. Geographically weighted regression did not reveal any local patterns in this association. Conclusions: Black–white racial disparities in CRC incidence were found in 28 counties in Mississippi. The county-level percentage of food insecurity emerged as a possible predictor of the observed black–white racial disparities in CRC incidence rates. Individual-level studies are needed to clarify whether food insecurity is a driver of these disparities or a marker of systemic disadvantage in these counties.

## Simple Summary

This ecological study investigated racial disparities in colorectal cancer (CRC) incidence rates between black and white populations in Mississippi counties from 2003 to 2020. The black population experienced significantly higher incidence rates than the white population in 28 counties of Mississippi. No hot spots were detected, indicating that there were no spatial clusters of areas with pronounced racial disparities. County-level food insecurity emerged as a potential predictor of the observed racial disparities in CRC incidence rates, suggesting a modifiable factor for policy interventions to reduce CRC disparities in Mississippi. Nonetheless, individual-level studies are necessary to determine whether food insecurity directly drives these disparities or is a marker of systemic disadvantage in these counties.

## 1. Introduction

Colorectal cancer (CRC) is the third most commonly diagnosed cancer in both men and women in the United States (U.S.) [1,2,3]. CRC ranks as the second most common cause of cancer-associated deaths among men aged 20 to 39 years and is the leading cause of cancer deaths in men under 50 [4]. The incidence of CRC increases significantly with age, rising from 33.1 per 100,000 among individuals aged 45–49 to nearly double that by ages 60–64 [4]. Black populations experience approximately 20% higher CRC incidence rates compared to non-Hispanic whites [5,6,7]. In the U.S., Mississippi reports the highest CRC incidence rates (48.2 per 100,000) and mortality rate (18 per 100,000) [5,8,9,10]. Mississippi also has substantial black and white populations, providing a unique opportunity to study racial disparities in CRC outcomes.

Recently, data on cancer incidence have become accessible for mapping cancer rates across the U.S. [11,12]. Unlike mortality data, which reflect both disease occurrence and treatment quality, the recent release of incidence data offers a more focused understanding of CRC patterns. A study conducted in North Carolina highlighted geographic differences in CRC incidence between black and white populations, revealing both elevated rates among black individuals and a hot spot in eastern North Carolina with pronounced racial disparities [13]. Given Mississippi’s high CRC incidence and its higher black population, we undertook a similar analysis. The study goal was to pinpoint areas where public health practitioners could focus efforts to address racial disparities in CRC incidence. Therefore, the objective of this study was to investigate county-level racial and geographical disparities in CRC incidence and the associated risk factors in Mississippi. This study’s findings can inform future research and intervention initiatives to decrease racial and geographic disparities in CRC incidence across Mississippi.

## 2. Materials and Methods

### 2.1. Study Design, Area, and CRC Data Sources

This retrospective ecological study was conducted in the counties of Mississippi. County-level CRC incidence and mortality data for black and white populations were sourced from the Mississippi Cancer Registry for the years 2003 to 2020 (https://www.cancer-rates.info/ms/, accessed on 9 March 2024). The cancer reporting system used in this study gathers data on the at-risk population, case counts, crude rates, age-adjusted rates, and 95% confidence intervals across all 82 counties in Mississippi. However, six counties were excluded from the disparity analyses due to small population sizes of either black or white individuals, which led to missing incidence rate data for one or both racial groups in these counties.

### 2.2. Predictor Variables

Data on potential predictors of CRC at the county level of Mississippi, including percent uninsured, percent smokers, percent diabetes, percent physical inactivity, percent obesity, percent food insecurity, and median income (Supplemental Table S1), were obtained from county health rankings, a program of the University of Wisconsin Population Health Institute and supported by the Robert Wood Johnson Foundation [14]. This program compiles health data from various sources, such as the U.S. Census Bureau and the American Community Survey. Cartographic boundary files were downloaded from the U.S. Census Bureau’s TIGER files and used for generating maps [15].

### 2.3. Descriptive Statistical Analysis

The difference in age-adjusted CRC incidence rates between black and white populations, along with 95% confidence intervals (CIs), was computed using MedCalc Statistical Software (version 22.021) [16]. We added the incidence rate difference and their 95% CIs to a Mississippi county shapefile and analyzed using ArcGIS Pro Version 2.7. The result was considered statistically significant if the 95% CI excluded zero.

### 2.4. Spatial Cluster Analysis

Using ArcGIS Pro, the Getis–Ord Gi* method was applied to detect statistically significant spatial clusters of the black–white CRC incidence rate difference at the county level. To control for the increased risk of false positives due to multiple comparisons (each county evaluated as a potential cluster center), *p*-values from the cluster analysis were adjusted using the false discovery rate (FDR) correction method [17]. Getis–Ord Gi* analysis identifies hot and cold spots [18]. Hot spots are areas where high values cluster, whereas cold spots are areas where low values cluster.

### 2.5. Regression Analysis

We initially ran a simple global ordinary least square (OLS) regression to investigate which factors were associated with racial differences in incidence rates. No variables were associated in simple regression models, but the percentage of food insecurity was the closest to being significant (Supplemental Table S2). Next, we created a post hoc directed acyclic graph (DAG) to visualize the relationships between the predictors and the outcome variable (black–white CRC incidence rate difference), with a focus on the percentage of food security as the primary predictor. We also generated a correlation matrix heatmap to provide a visual representation of the pairwise correlations between predictor variables (Supplemental Figure S2). Utilizing this correlation matrix heatmap, we identified the percentage of smoking variable as highly correlated with the percentage of food insecurity, and consequently, we excluded the percentage of smoking variable from our analyses to mitigate potential multicollinearity issues.

Our primary OLS regression analysis used a continuous outcome of racial differences in incidence rates. In a secondary analysis, because some areas have large racial differences but small sample sizes, we ran a logistic regression based on areas that had significant racial disparities in incidence rates (yes/no). For each global multiple regression analysis, we adjusted the covariates identified in the DAG (Supplemental Figure S1) using three statistical models: Model 1: unadjusted, including only percentage of food insecurity and outcome variable; Model 2: adding potential confounding variables (percentage of uninsured and median income) associated with both exposure and outcome variables; Model 3: full model including the exposure and all other covariates identified in the DAG (percentage of food insecurity, percentage of uninsured, median income, percentage of obesity, percentage of physical inactivity, and percentage of diabetes). We also checked for possible multicollinearity using the variance inflation factor (VIF) to avoid issues associated with multicollinearity for the regression models, ensuring that the VIF value did not exceed 5 in any of our models [19]. In addition, we evaluated the Akaike Information Criterion (AIC) for the three models in each multiple regression analysis. The model with the lowest AIC values was considered to provide the best fit and parsimony compared to the other models, and we interpreted this model output in this study. The beta coefficient (β) and 95% confidence intervals (CIs) were reported for the exposure variable from the OLS regression models. The odds ratios (ORs) and 95% confidence intervals (CIs) were reported for the exposure variable from the logistic regression models.

As sensitivity analyses, we conducted multiple ordinal logistic regression to examine the association between the percentage of food insecurity and the quartiles of black–white CRC incidence rate difference (outcome of interest). We also investigated whether results were consistent using mortality rate difference as our dependent variable. The Brant test was performed after fitting the final multiple ordinal logistic regression model to assess whether the proportional odds assumption holds. A non-significant *p*-value (*p* > 0.05) indicates that the proportional odds assumption holds.

We also conducted local geographically weighted multiple regression (GWR) models to identify global and locally varying predictors associated with differences in CRC incidence rates, and logistic GWR models for investigating counties exhibiting significant racial disparities in CRC incidence rates. The local OLS and logistic GWR models were implemented in the MGWR version 2.2 software [20]. The estimation of the local GWR coefficient was based on the spatial kernel adaptive bi-square method.

The global Moran’s I test was conducted to assess global spatial dependence (autocorrelation) in the residuals of the global OLS model using queen spatial weights, implemented in GeoDa Version 1.22. The null hypothesis was that residuals were randomly distributed across the study area. The significance test of global Moran’s I was conducted using GeoDa’s randomization of 999 permutations; a *p*-value less than the cut-off 0.05 was regarded as a significant result and would necessitate corrections in the regression model to account for the spatial dependence in the residuals [21].

### 2.6. Cartographic Display

**Figure 1:**
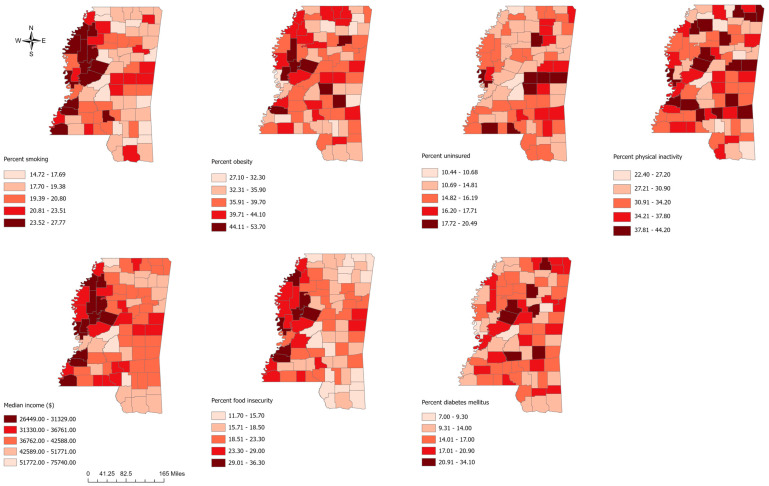
Geographic distribution of potential predictors (percent smoking, percent obesity, percent uninsured, percent physical inactivity, median income, percent food insecurity, and percent diabetes mellitus) of county-level colorectal cancer incidence rate difference per 100,000, between black and white populations in Mississippi counties, 2003–2020.

All maps were generated using ArcGIS Pro software (version 2.7; ESRI, Redlands, California, https://www.esri.com/en-us/arcgis/products/arcgis-pro/overview, accessed on 25 March 2024). Choropleth maps were used to display the geographic distribution of the age-adjusted incidence rate of CRC among the black and white populations, CRC incidence rate differences between black and white populations, and possible predictor variables (Figure 1). Critical intervals for the choropleth map were determined using a natural breaks (Jenk’s optimization) classification scheme [22].

## 3. Results

### 3.1. Descriptive Statistics

**Figure 2:**
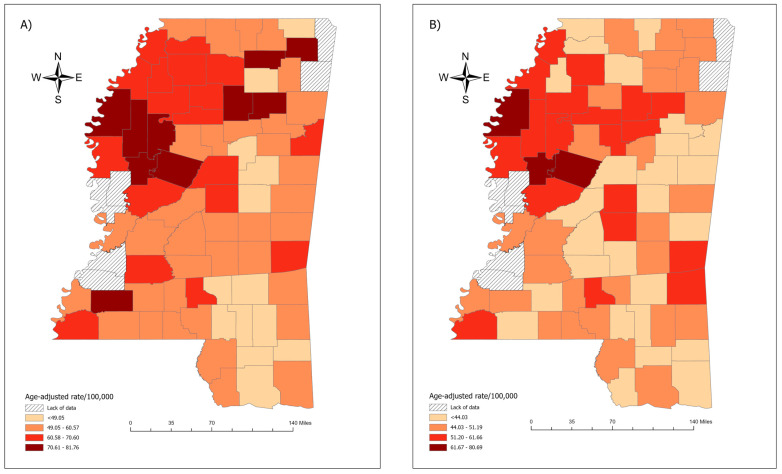
Colorectal cancer incidence rates (2003–2020) among black and white populations by county. Data were obtained from the Mississippi Cancer Registry: (**A**) age-adjusted incidence rate (per 100,000) of colorectal cancer among the black population at the county level; (**B**) age-adjusted incidence rate (per 100,000) of colorectal cancer among the white population at the county level.

The age-adjusted CRC incidence rate in the black population varied by geographical area, ranging from 31.81 to 81.76 per 100,000 population (median = 58.12/100,000 population). The highest age-adjusted CRC incidence rates in the black population were observed in the northwest and northeast counties of Mississippi (Figure 2A). Likewise, geographic patterns of age-adjusted CRC incidence rates in the white population varied by geographical area, ranging from 35.29 to 80.69 per 100,000 population (median = 46.44/100,000 population). The highest age-adjusted CRC incidence rates in the white population were observed in the northwestern counties of Mississippi (Figure 2B).

**Figure 3:**
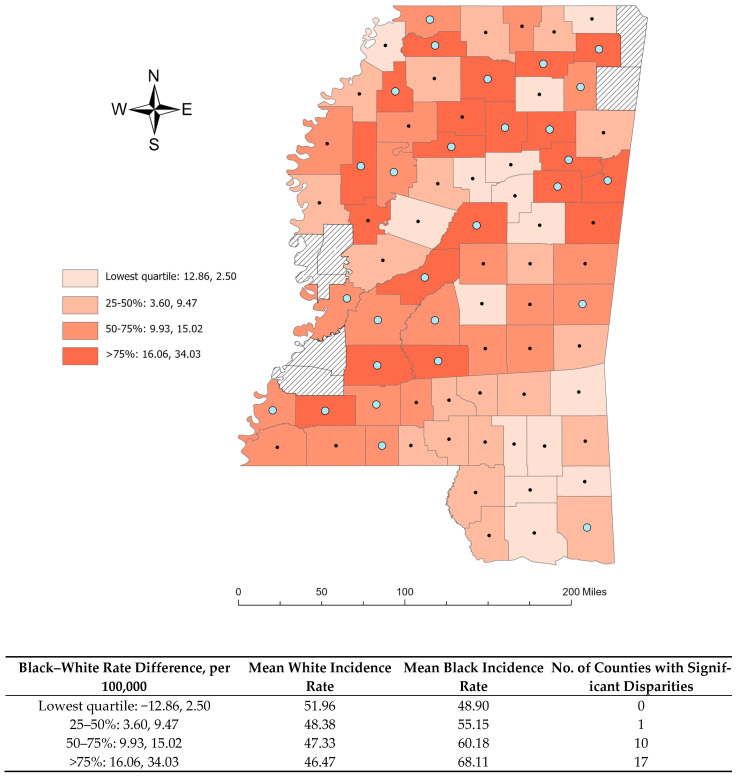
Colorectal cancer incidence rate differences per 100,000, between black and white populations in Mississippi counties, 2003–2020. Counties with significant rate differences (RDs) are indicated with a wider blue circle 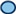.

The differences in CRC incidence rates between the black population and white populations across Mississippi are visualized in Figure 3. Statistically significant racial differences in CRC incidence rates were identified in 28 counties, all of which showed higher incidence rates among the black population compared to the white population. While a few counties exhibited higher incidence rates among the white population compared to the black population, these differences were not statistically significant.

The ethnic population in Mississippi was predominantly non-Hispanic white and non-Hispanic black. The non-Hispanic white population had a median representation of 57.27% (range: 15.72% to 86.80%), while the non-Hispanic black population had a median of 37.41% (range: 7.81% to 81.95%) (Supplemental Table S3).

### 3.2. Spatial Clustering Results

The Getis–Ord Gi* analysis did not reveal any hot or cold spots, suggesting the absence of concentrated clusters of racial disparities. The disparities appeared to be dispersed across various areas of the state.

### 3.3. Regression Analysis Results

We explored whether county-level factors might be associated with racial differences in CRC incidence rates. Our initial global OLS simple regression models suggested that the percentage of food insecurity had the strongest association with the CRC incidence rate differences (Supplemental Table S2). Our post hoc DAG guided the selection of our three regression models (unadjusted, minimally adjusted, and fully adjusted). The unadjusted global OLS model did not show an association between the percentage of food insecurity and racial differences in CRC incidence (β = 0.36; 95% CI: −0.08, 0.79). Our parsimonious model, which adjusted for median income and percent uninsured showed a significant association (β = 0.93, 95% CI: 0.25, 1.62), as did the fully adjusted model (β =1.03, 95% CI: 0.26, 1.81) (Table 1). The parsimonious model had the lowest AIC value and is interpreted to show a 1% increase in food insecurity associated with a 0.93/100,000 differential increase in the black-white CRC incidence rate. Global Moran’s I of the residuals (Moran’s I = 0.061, *p* = 0.13) was not significant, indicating no need to account for spatial dependence of the residuals. Additionally, the GWR multiple regression results show that the percentage of food insecurity was positively associated with the CRC incidence rate difference (mean β = 0.547, range: 0.269 to 0.802) across counties in Mississippi, without significant spatial variability in the coefficients of the percentage of food insecurity (*p* = 0.32). Additionally, no multicollinearity was found in the multiple OLS regression model (Supplemental Table S4).

Using significant/non-significant differences in CRC incidence rates as the dependent variable to better account for small sample sizes in some counties with large racial disparities, our global multiple logistic regression model found similar results of an association between percent food insecurity and racial differences in CRC incidence, significant with the smallest AIC in the parsimonious model (OR = 1.25; 95% CI:1.02, 1.52) (Table 1). Additionally, the GWR multiple logistic regression results show that the percentage of food insecurity was positively associated with the racial differences in CRC incidence across counties (mean β = 1.35, range: 1.18 to 1.55) in Mississippi, without significant spatial variability in the coefficient for the percentage of food insecurity (*p*= 0.96).

In a sensitivity analysis, using quartiles of black–white colorectal cancer incidence rate difference as the outcome variable in a multiple ordinal logistic regression model, we found generally similar results. We identified a 1% increase in food insecurity associated with 19% higher odds of being in a higher quartile of CRC incidence rate difference (OR = 1.19, 95% CI: 1.03, 1.36) in the parsimonious model, although the unadjusted model had the lowest AIC, and those results were not significant (OR = 1.07; 95% CI: 0.98, 1.16) (Table 1).

In another sensitivity analysis that focused on mortality instead of incidence, we used quartiles of black–white colorectal cancer (CRC) mortality rate difference as the outcome variable. The parsimonious multiple ordinal logistic regression model identified an association between every 1% increase in food insecurity and 30% higher odds of being in a higher quartile of the black–white CRC mortality rate difference (OR = 1.30, 95% CI: 1.11, 1.51; Supplemental Table S5).

## 4. Discussion

In Mississippi, the black population had significantly higher CRC incidence rates than the white population in 28 counties. Our analysis revealed no hot spots, suggesting that racial disparities were not localized within specific clusters. Between 2003 and 2019, Mississippi’s overall CRC incidence rate (52.24 per 100,000) exceeded the national average of 36 per 100,000 [23]. In this study, the CRC incidence rate among the black population was 58.12 per 100,000, compared to 46.44 per 100,000 among the white population.

We explored whether county-level community health factors might be associated with black–white differences in CRC incidence rates. Our findings provide some evidence that food insecurity may be linked to the black–white differences in CRC incidence rates across Mississippi counties. As a social determinant of health, food insecurity can result in inadequate intake of essential nutrients, which may play a role in colorectal health. Food insecurity may contribute to the increased risk of CRC through dietary patterns. A study revealed that individuals experiencing food insecurity were more likely to consume red meat and processed red meat prepared using high-temperature cooking methods such as pan-frying and grilling [16]. These practices have been linked to carcinogens, including 2-amino-3,8-dimethylimidazo[4,5-f]quinoxaline (MeIQx), 2-amino-3,4,8-trimethylimidazo[4,5-f]quinoxaline (DiMeIQx), and benzo[α]pyrene (BaP)], which may contribute to an increased risk of CRC compared to food-secure individuals [24]. Addressing dietary patterns within communities could mitigate the risk of CRC. For example, diets rich in whole grains, vegetables, fruit, and dairy products and low in red meat and processed meat have been associated with a reduced risk of CRC [25]. Also, the chronic stress associated with food insecurity may weaken the immune system and potentially increase susceptibility to cancer [26,27]. Interestingly, other county-level social determinants of health, including median income, percent uninsured, diabetes, obesity, and physical inactivity, were not associated with racial disparities in CRC incidence in our study.

While county-level percentage of food insecurity was identified as a significant factor associated with black–white CRC incidence rate racial disparities, it may reflect broader socioeconomic factors that contribute to black–white CRC incidence rate racial disparities at the individual level. The present study is an ecological investigation, wherein estimates of risk factors and cancer rates are only available at the county level. This research cannot be directly applied to individual-level variables associated with CRC incidence rate differences to avoid the ecological fallacy [28,29]. Future research should aim to clarify the underlying drivers of these racial disparities in CRC incidence using individual-level data.

Our study has several strengths. This is the first study to investigate the spatial patterns in racial disparities in CRC incidence in Mississippi, a state with the highest rates of CRC incidence and mortality in the U.S. [5,8]. We identified areas with significant racial disparities in CRC incidence and explored whether county-level risk and protective factors were associated with the observed racial disparities. We conducted global and local regression analyses to characterize the associations, both overall and in local parts of the state. We defined the outcome variable in two ways: as a continuous black–white incidence rate difference and as a categorical definition of racial disparity based on the presence or absence of significant racial disparities in a county. We also conducted sensitivity analyses utilizing quartiles of black–white CRC incidence and mortality rate differences as outcome variables, and the overall consistent findings indicated that the percentage of food insecurity at the county level may be associated with black–white CRC racial disparities in Mississippi. Nevertheless, individual-level studies are needed to confirm any links between food insecurity and racial disparities in CRC.

Our study has several limitations that are common to ecological studies. For example, we did not have individual-level data on cancer outcomes or risk/protective factors, and not all factors were available at the county level for our analysis. Preventive colonoscopies and sigmoidoscopies can remove polyps before turning into cancer, and black residents of Mississippi reported a higher rate of never having undergone a colonoscopy or sigmoidoscopy (53.2%) compared to white residents (41.9%) [30]. Unfortunately, we were unable to identify county-level data on preventive colonoscopies/sigmoidoscopies. Future studies incorporating stage-specific and colonoscopy rate data could offer valuable insights into how these factors influence racial disparities in CRC outcomes at different stages. We also recognize that individual-level genetic markers such as Tob1 gene expression and individual-level risk factors such as diet and alcohol consumption could play a role in understanding CRC disparities; hence, future individual-level investigations should include Tob1 gene expression and individual-level risk factors that may influence racial disparities in CRC. Furthermore, counties with small populations may skew results in logistic regression models. In the regression analyses, we excluded 6 out of 82 counties from the disparity analyses due to the small population sizes of either black or white individuals. In addition, we conducted linear and ordinal regression analyses, including sensitivity analysis with CRC mortality as an outcome, which consistently identified food insecurity as a potential predictor of the observed CRC incidence rate disparities. Additionally, we lacked sufficient statistical power to stratify the analysis by sex or cancer stage, thus limiting our ability to examine racial disparities within these subgroups. In addition, our study focused on disparities in CRC incidence only between black and white populations because there was only black and white race information in the Mississippi State Cancer Registry database. However, this approach could be adopted to compare disparities across more than two racial/ethnic groups. In states with sufficient populations of Hispanics or other races, a similar approach could be used to clarify racial disparities in CRC among other groups.

## 5. Conclusions

In conclusion, our study revealed black–white racial disparities in CRC incidence in 28 counties in Mississippi. Food insecurity emerged as a possible predictor of the observed CRC incidence rate differences. The potential link between food insecurity and black–white racial disparities in CRC incidence rates is a modifiable factor that could guide policy interventions to reduce CRC disparities in Mississippi. However, future individual-level studies are needed to clarify whether food insecurity is a driver of these disparities or a marker of systemic disadvantage in these counties, in an effort to map a path for mitigating these disparities.

## Figures and Tables

**Table 1:** Adjusted coefficients and odds ratios for the association between the percentage of food insecurity and outcome variables (OLS regression outcome: black–white colorectal cancer incidence rate differences; logistic regression outcome: significant black–white colorectal cancer incidence rate differences; ordinal logistic regression outcome: quartiles of black–white colorectal cancer incidence rate difference) at the county level in Mississippi using three different global multiple regression models.

Exposure Variable	Model 1 (Unadjusted)	Model 2 (Minimally Adjusted)	Model 3 (Fully Adjusted)
Multiple OLS regression			
% Food Insecurity (β, 95% CI)	0.36 (−0.075, 0.79)	0.93 (0.25, 1.62)	1.03 (0.26, 1.81)
AIC	555.12	554.41	557.89
Multivariable Logistic Regression			
% Food Insecurity (OR, 95% CI)	1.00 (0.91, 1.10)	1.25 (1.02, 1.52)	1.31 (1.05, 1.64)
AIC	104.03	93.32	96.81
Multivariable Ordinal Logistic Regression			
% Food Insecurity (OR, 95% CI)	1.07 (0.98, 1.16)	1.19 (1.03, 1.36)	1.22 (1.04, 1.42)
AIC	216.35	216.57	217.07

Footnotes: β = beta coefficient, OR = odds ratio, CI = confidence interval, OLS regression models: coefficients (β) represent the change in Black-White colorectal cancer incidence rate difference (per 100,000) associated with a 1% increase in food insecurity. For example, a β value of 0.93 for % food insecurity indicates that a 1% increase in food insecurity is associated with a 0.93/100,000 differential increase in the black–white colorectal cancer incidence rate. Logistic regression models: odds ratios (ORs) represent the odds of significant black–white colorectal cancer incidence rate differences occurring for a 1% increase in food insecurity. For instance, an odds ratio of 1.25 for % food insecurity indicates that for each 1% increase in food insecurity, the odds of the areas of significant black–white colorectal cancer incidence rate difference occur 1.25 times higher. Ordinal logistic regression models: odds ratios (ORs) represent the odds of being in a higher quartile of black–white colorectal cancer incidence rate difference for a 1% increase in food insecurity. For example, an odds ratio of 1.19 for % food insecurity indicates that each 1% increase in food insecurity is associated with 19% higher odds of being in a higher quartile of colorectal cancer incidence rate difference. Model adjustments: Model 1 (Unadjusted) included only % food insecurity as a predictor of interest. Model 2 (minimally adjusted) was adjusted for median income and % uninsured. Model 3 (fully adjusted) was adjusted for % uninsured, median income, % obesity, % physical inactivity, and % diabetes. AIC: Akaike Information Criterion; lower AIC indicates a better model fit. Brant test (ordinal logistic models): *p* = 0.992; proportional odds assumption holds for the ordinal logistic model.

## Data Availability

Data are publicly available from the Mississippi Cancer Registry from 2003 to 2020 (https://www.cancer-rates.info/ms/, accessed on 9 March 2024).

## Supplementary Materials

The following supporting information can be downloaded at: https://www.mdpi.com/article/10.3390/cancers17020192/s1, Figure S1: Directed acyclic graph (DAG) showing the hypothesized association between % food insecurity (exposure) and significant Black-White colorectal cancer incidence rate difference (outcome) with covariates; Figure S2: Correlation Matrix Heatmap of Key Predictor and Covariates; C_corr: correlation coefficient values (r); pct_foodin (% food insecurity), pct_uninsu (% uninsured); med_income (median income), pct_adultD (%Diabetes); pct_obesit (%obesity), pct_phylna (%physical inactivity), pct_smoke (%smoking). Table S1: Source and description of predictors at the county level in Mississippi, obtained from county health rankings, a program of the University of Wisconsin Population Health Institute and supported by the Robert Wood Johnson Foundation; Table S2: Univariable (unadjusted) association between predictors and racial differences in colorectal cancer (CRC) incidence rates: results from univariable OLS linear regression model; Table S3: Ethnic population distribution in Mississippi; Table S4: Variance Inflation Factor (VIF) for each predictor; Table S5: Adjusted odds ratio (OR) for quartiles of black-white colorectal cancer (CRC) mortality rate difference and the percentage (%) of food insecurity at the county level in Mississippi, using multiple ordinal logistic regression models.
