# Supplementary material for: Racial and Geographic Disparities in Colorectal Cancer Incidence and Associated County-Level Risk Factors in Mississippi, 2003–2020: An Ecological Study
Source: Cancers (Basel). 2025 Jan 9;17(2):192. doi: 10.3390/cancers17020192 (PMC11764440; doi:10.3390/cancers17020192)
Supplement: Supplementary file 1 [file cancers-17-00192-s001.zip › cancers-3382011-supplementary.pdf]

Table S1: Source and description of predictors at the county level in Mississippi, obtained from county health rankings, a program of the University of Wisconsin Population Health Institute and supported by the Robert Wood Johnson Foundation

**Predictor**	**Description and measure**	**Source**	**Year**
%Uninsured	Percentage of adults under age 65 without health insurance	Small Area Health Insurance Estimates	2017
%Food insecure	Percentage of population who lack adequate access to food	Map the Meal Gap	2017
%Smokers	Percentage of adults who are currently smokers	Behavioral Risk Factor Surveillance System	2017
%Diabetes	Percentage of adults aged 20 and above with diagnosed diabetes	United States Diabetes Surveillance System	2016
Median household Income	The income where half of households in a county earn more and half of households earn less	Small Area Income and Poverty Estimates	2018
% Obesity	Percentage of adult populations (age 20 and older) that reports a body mass index (BMI) greater than or equal to 30 Kg/m2	United States Diabetes Surveillance System	2016
%Physical inactivity	Percentage of adults age 20 and over reporting no leisure-time physical activity	United States Diabetes Surveillance System	2016

Table S2: Univariable (unadjusted) association between predictors and racial differences in colorectal cancer (CRC) incidence rates: results from univariable OLS linear regression model

Predictor	Coefficient (β)	p-value	95% Confidence Interval
% Food insecurity	0.359	0.104	-0.071, 0.079
% Uninsured	0.059	0.920	-1.10, 1.23
Median income ($1,000)	-0.002	0.986	-0.254, 0.249
% Diabetes	-0.236	0.369	-0.757, 0.284
% Obesity	0.037	0.880	-0.459, 0.535
% Physical inactivity	-0.294	0.188	-0.736, 0.147

Footnotes:

Dependent Variable: Racial differences in CRC incidence rates (Black-White CRC incidence rate difference).

Univariable (Unadjusted) OLS Linear Regression: Each predictor is analyzed individually to examine its association with racial differences in incidence rates.

Coefficient (β): This represents the change in the incidence rate difference for a 1-unit increase in the predictor variable.

Table S3: Ethnic population distribution in Mississippi

Race	Description	Median (Range)	Source	Year
% Non-Hispanic Black	Percentage of population that is non-Hispanic Black or African American	37.41 (7.81, 81.95)	Census Population Estimates	2018
% American Indian & Alaska Native	Percentage of population that is American Indian or Alaska Native	0.34 (0.10, 19.19)	Census Population Estimates	2018
%Asian	Percentage of population that is Asian	0.43 (0.08, 3.11)	Census Population Estimates	2018
%Native Hawaiian/other pacific Islander	Percentage of population that is Native Hawaiian or Other Pacific Islander	0.03 (0, 0.44)	Census Population Estimates	2018
% Hispanic	Percentage of population that is Hispanic	2.14 (0.93, 11.51)	Census Population Estimates	2018
%Non-Hispanic White	Percentage of population that is non-Hispanic White	57.27 (15.72, 86.80)	Census Population Estimates	2019

Table S4: Variance Inflation Factor (VIF) for each predictor

Variable	VIF
Median income	4.14
%Food insecurity	3.27
%Uninsured	1.77
%Diabetes	1.66
%Physical inactivity	1.54
%Obesity	1.47
Mean VIF	2.31

Footnotes: VIF > 5: A VIF value greater than 5 indicates a high degree of multicollinearity.

Table S5: Adjusted odds ratio (OR) for quartiles of black-white colorectal cancer (CRC) mortality rate difference and the percentage (%) of food insecurity at the county level in Mississippi, using multiple ordinal logistic regression models.

**Exposure variable**	**Model[1]**	**Model[2]**	**Model[3]**
	**OR (95% CI)**	**OR (95% CI)**	**OR (95% CI)**
% food insecurity	1.12 (1.02, 1.22)	1.30 (1.11, 1.51)	1.34 (1.13, 1.60)
AIC	203.86	201.40	205.74

Footnotes:

OR=odds ratio, CI=confidence interval, AIC= Akaike Information Criterion.

Brant Test: p = 0.077; the proportional odds assumption holds, so the multivariable ordinal logistic regression model is appropriate.

Model[1](Unadjusted model) includes % food insecurity (continuous).

Model[2] (minimally adjusted model) includes % food insecurity (continuous), median income (continuous), % uninsured (continuous);

Model[3] (fully adjusted model) includes % food insecurity (continuous), median income (continuous), % uninsured (continuous), , % obesity (continuous), % physical inactivity (continuous), and % diabetes (continuous).

Interpretation of OR: The odds ratios indicate the change in odds of the outcome variable (quartiles of black-white colorectal cancer mortality rate difference) occurring for a 1% increase in the corresponding predictor variable. For example, an odds ratio of 1.30 for % food insecurity indicates that each 1% increase in food insecurity is associated with 30% higher odds of being in a higher quartile of black-white colorectal cancer mortality rate difference.

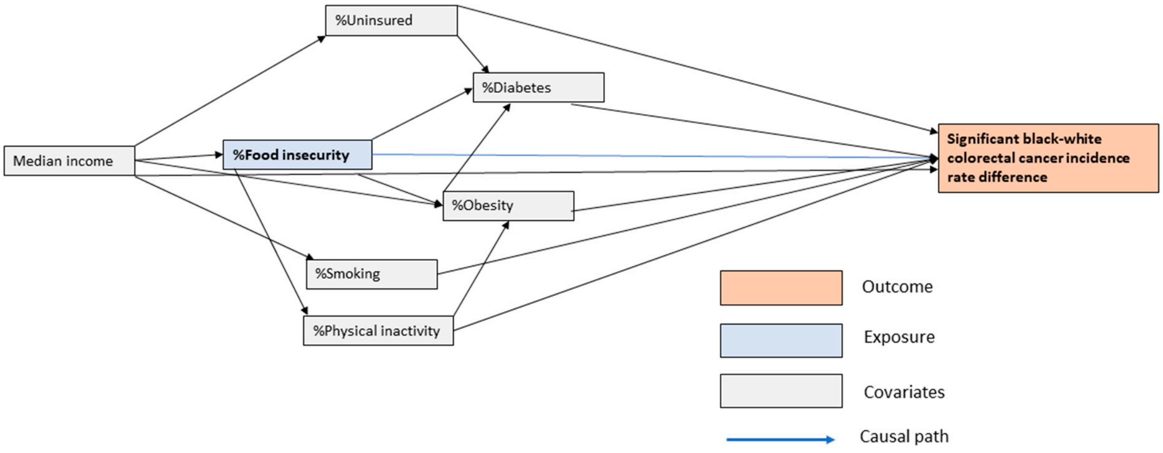

Figure S1: Directed acyclic graph (DAG) showing the hypothesized association between % food insecurity (exposure) and significant Black-White colorectal cancer incidence rate difference (outcome) with covariates.

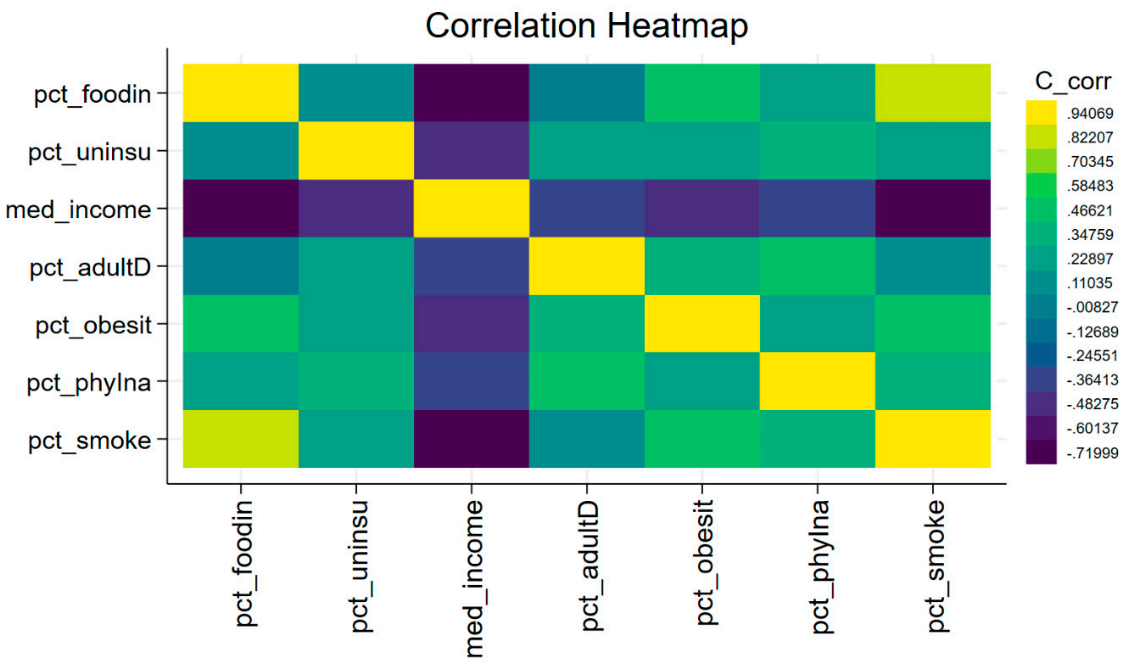

Figure S2: Correlation Matrix Heatmap of Key Predictor and Covariates; C_corr: correlation coefficient values (r); pct_foodin (% food insecurity), pct_uninsu (% uninsured); med_income (median income), pct_adultD (%Diabetes); pct_obesit (%obesity), pct_phylna (%physical inactivity), pct_smoke (%smoking).
